# Editorial

**Published:** 1903-09-15

**Authors:** 


### EDITORIAL.

THE new college year is about to commence and it will be the beginning of a new era in dental instruction. Although no innovations are to be expected in the methods of imparting instruction and very few at present in the curriculum, it means that a serious effort is to be made to increase in breadth and quality the course of instruction. The sphere of the dentist's occupation has increased considerably since the three years' course of instruction was generally adopted, and the high order of skill required to assure success in these days demands an amount of culture and skill that the three years' course could not attain, except that a higher and different order of preliminary training was exacted for admission. And since it seems impracticable to secure in the secondary or preparatory schools the proper training to enable the dental colleges to dispense with fundamental studies and technical training, it has become imperative that more time shall be spent in dental colleges, where the student's entire professional training may be developed in a normal and practicable manner. And it is just at this point that those who are seeking to increase the value of professional culture by advocating the academic degree for admission to the dental schools and a shorter course in the dental school are probably making a mistake. The academic degree means more mental training and is certainly to be desired, but it also means three or four of the best years are lost for technical training, and this opportunity never comes again, as the high-school graduate has seldom passed his eighteenth birthday and has not passed beyond the plastic or impressionable stage of his existence, but he has reached a point where he begins to think seriously of his life work and to make suitable preparations for it. It would seem that we are justified in taking this step forward and we ought to expect its full consummation to bring forth results commensurate to the sacrifice of time involved.

—✦—

The National Association of Faculties, National Association of Examiners and National Dental Association all convened as advertised at Asheville, N. C. The place was all that had been promised. The weather was comfortable and the welcome as cordial as traditional in that latitude. The Faculties Association began its session on Friday, July 24th, and transacted the usual routine business. No specially important business of interest to the profession was considered except that the Dental Department of Harvard University declined to go with the Association to a four years' course of study, and withdrew its membership from the Association. The other schools in the Association declined to be influenced by this action and will stand by the action establishing this course. There was considerable discussion of the subject and effort was made to convince the Harvard representative that it was desirable to carry out this idea. The action of Harvard is based on the fact that this school will very soon require an academic degree for admission to its professional course. There was considerable discussion in regard to advanced standing given to graduates of foreign schools who may wish to take the American degree. The Foreign Relations Committee recommended two years ago that not more than one year's credit be allowed any foreign school and this recommendation was approved by the Association. This action has not met the approval of several of the larger Eastern schools who have these students applying for admission every year, and the protest resulted this year in a reogranization of this committee which will probably mean a change of action on this matter. There is no question but that some of the best schools of the old countries graduate men well qualified theoretically, in the scientific branches, but they are notably weak in technical training, and one year of residence in an American school is not believed to be sufficient to overcome this defect. It is also to be regretted that these foreign schools will not recognize any part of the instruction given in our schools. A mutual and reciprocal action on this subject would doubtless be better for all concerned.

The Association seems to be getting very particular as to its membership. It declined the petition for membership of a school which has sprung up as a department of a medical college. If this idea should become retroactive we fear there would be only a select few in the Association's membership.

The officers elected for the ensuing year are M. C. Marshall, of St. Louis, President; E. C. Kirk, of Philadelphia, Vice-President; J. H. Kennerly, of St. Louis, Secretary; H. R. Jewett, of Atlanta, Treasurer. The Association will probably meet at St. Louis, next year.

The meetings of the National Association of Examiners are executive sessions and no report of its actions can be obtained. It is said that the subject of interstate reciprocity was the most important subject discussed. It would not be a bad idea if this body would put its wits to work in devising a dental law which would be equitable in action, and that could be enacted into the statutes of every State, so that by it a uniform standard of attainment might be had in the whole country. If this could be done interstate reciprocity would come without trouble.

The National Association is of course the most important and largest attended meeting of the group. About six hundred dentists attended this year and the accommodations of the hotel were severely taxed. The resident dentists were hospitable and did much to make the stay enjoyable by trip to the mountain top and "Biltmore," the country place of Mr. George Vanderbilt. The papers read were interesting and valuable. As in most societies during the past year, the subject of porcelain inlays seemed to have the precedence in number and interest. As is always the case in this meeting, there are too many papers and too little discussion, or at least, a few papers seem to get all the attention and many others are either read by title or get little or no public consideration. The constitution was amended so that hereafter the papers will be read before three sections meeting simultaneously. This plan will permit of three times as much work being done, but one will need to choose the section he shall give his time to, as he can't very well be in three places at one time. The clinics were unusually interesting and the whole meeting successful. The meetings will hereafter be held commencing the last Tuesday in July. The next meeting will however be held in connection with the St. Louis World's Fair, and will be an International Dental Congress. This will be the fourth in the series, and active preparations are already under way to make it represent the present status of dentistry the world over. The general committee of organization has already in outline decided on the general features to be presented, and is at work appointing its sub-committees for the detail work. It is expected that this will be the most largely attended dental meeting ever held.

The officers elected were, C. C. Chittenden, of Wisconsin, President; A. H. Peck, Chicago, Secretary; V. E. Turner, Raleigh, N. C., Treasurer.

A meeting of the Dental Protective Association was held at which a statement of the Association's affairs was made and of the work done and to be done by the Association.

There was also held the annual meeting of Delta Sigma Delta Fraternity, and a banquet was given by the new Interstate Dental Fraternity, an organization which is to meet once each year its ritual and object seem to be all centered in a supper and speeches.

—✦—

BELOW we print a list of the numbers graduated by our dental schools last year. It would seem that more than two thousand new dentists ought to be able to take good care of the increasing population, and fill the places of those who have dropped out for various reasons. These men are well prepared for their work, and the enlightened public will make good use of every earnest and faithful man of them. It is to be hoped that they all have the true idea of their mission. We fear some will forget that they are called to this work as truly as the clergyman is to the more spiritual ministry. It would be a very helpful idea if each one could feel like Hiram Golf, who spoke of himself as "a shoemaker by the grace of God," and believe that dentistry was as exalted and noble a calling as many others which are seemingly more honored. There will be no lack of opportunity for services if it can be rendered by one with such an ideal.

—✦—

List of the number of graduates from each dental college for the last session:

Atlanta Dental College, 46; Baltimore College of Dental Surgery, 57; Baltimore Medical College, 23; Birmingham Dental College, 4; Central Indiana Dental College, 4; Chicago College of Dental Surgery, 167; Cincinnati College of Dental Surgery, 15; Colorado College of Dental Surgery, 19; Columbian University, 7; Des Moines College of Dental Surgery, 19; Detroit College of Medicine, 46; Georgetown University, —; Harvard University, 27; Howard University, 9; Indiana Dental College, 65; Kansas City Dental College, 28; Keokuk Dental College, 19; Lincoln Dental College, 9; Louisville College of Dental Surgery, 65; Marion-Sims Dental College, 39; Medico-Chirurgical College, 24; Meharry Medical College, 5; Michigan University, 81; Milwaukee Medical College, 32; Missouri Dental College, 30; National University, 8; New Orleans College of Dentistry, 15; New York College of Dentistry, 54; New York Dental School, 12; North Pacific Dental College, 23; Northwestern University, 175; Ohio

College of Dental Surgery, 81; Ohio Medical University, 62; Pennsylvania College of Dental Surgery, 80; Philadelphia Dental College, 120; Richmond University, 10; Royal College of Dental Surgeons, 54; San Francisco College of Physicians and Surgeons, 22; San Francisco Dental College, 4; Southern Dental College, 32; Tuft's College, 48; University of Buffalo, 75; University of California, 43; University of Southern California, 16; University of Illinois, 40; University of Iowa, 32; University of Maryland, 64; University of Minnesota, 34; University of Omaha, 32; University of Pennsylvania, 102; University of Tennessee, 16; Vanderbilt University, 18; Western Dental College, 60; Western Pennsylvania University, 57; Western Reserve University, 40; Wisconsin College of Physicians and Surgeons, 6.

Total, 2,295.

